# Application of a Foot Plate in the Taylor Spatial Frame for the Treatment of Acquired Equinus or Equinovarus Deformities

**DOI:** 10.7759/cureus.84763

**Published:** 2025-05-25

**Authors:** Thylane E Vancastell, Sander V Ianniello, Kareem Abdelhakim-Edres, Joseph Muscat, Matija Krkovic

**Affiliations:** 1 Trauma and Orthopedic Surgery, Addenbrooke's Hospital, Cambridge University Hospitals National Health Service (NHS) Foundation Trust, Cambridge, GBR; 2 Trauma and Orthopedics, The Queen Elizabeth Hospital King's Lynn National Health Service (NHS) Foundation Trust, King's Lynn, GBR; 3 Trauma and Orthopedics, Addenbrooke's Hospital, Cambridge University Hospitals National Health Service (NHS) Foundation Trust, Cambridge, GBR

**Keywords:** ankle deformity correction, complications in taylor-spatial-frames, equinus deformity, fine wire frame, footplate, foot plate, lower leg deformity correction, lower limb fractures, taylor-spatial-frame, tsf

## Abstract

Fine wire frames are extensively utilized in the management of complex lower limb fractures and for deformity correction. Patients may develop equinus or equinovarus deformities in their feet due to the intricacies associated with the treatment. This may occur due to an inability to exercise and bear weight on the foot for various reasons or, more commonly, as a consequence of reverse tibial bone transport, in which the corticotomy is performed at the distal fragment, resulting in proximal bone transport. This technical note delineates the application of a foot plate to an existing fine wire frame when conservative management of equinus deformity of the foot has proven inadequate. The primary advantage of this technique lies in its capacity to facilitate a gradual increase in the movement of the ankle joint, all while being relatively minimally invasive. Our preliminary findings suggest that this method is safe and effective; however, it is imperative to note that there is a relatively high risk of recurrence of the deformity if the regained range of movement is not adequately protected through bracing or physiotherapy following the removal of the foot plate.

## Introduction

Fine wire frames are highly versatile surgical instruments in many complex cases, including deformity corrections and open fractures with bone defects [1]. In our practice, certain patients with complex injuries, primarily due to uncontrollable pain, develop equinus in the affected foot. Additionally, there exists a cohort of patients undergoing retrograde tibia bone transport, wherein corticotomy is performed at the distal fragment of the tibia and subsequently transported proximally. These individuals initially manifest equinus, which is thereafter followed by the development of equinovarus deformity. This phenomenon appears consistent in most reverse bone transport patients and cannot be mitigated through exercise [2-4]. However, if we intervene promptly, the onset of this deformity may be prevented by applying a foot plate. The foot plate itself significantly impacts the function of the limb and exacerbates pain. The primary challenge in retaining movement within the ankle joint is controlling the foot for exercise. When patients cannot walk, typically due to pain, it becomes nearly impossible to maintain an adequate range of motion in the ankle joint. Applying a foot plate provides substantial control over the foot, facilitating improvements in varus and equinovarus deformity.

## Technical report

**Figure 1:**
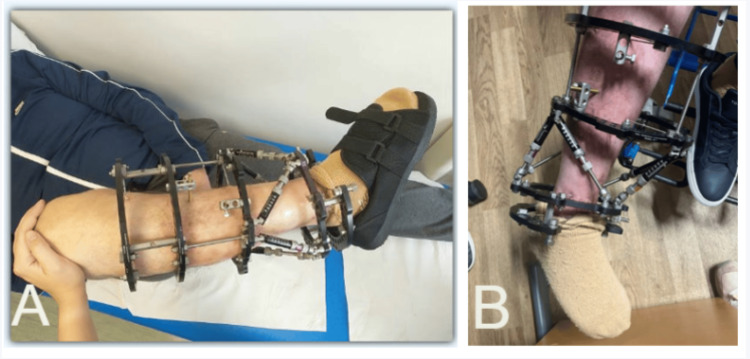
Photographic imaging of a fixed equinovarus deformity in a pre-existing Taylor spatial frame on the right lower leg. A: Lateral view. B: Anterior view.

Various criteria exist for assessing the normal dorsiflexion of the foot. In our practice, we maintain that as soon as a patient cannot achieve a plantigrade position, an equinus deformity is present (Figure 1).

We tend to address this condition promptly rather than delaying intervention. Standard treatment methods include lengthening the tibialis posterior and Achilles tendon [5,6]. When a fine wire frame is present on the same leg, the procedure may prove technically challenging, if not impossible. Furthermore, the aforementioned procedures are associated with an increased risk of soft tissue complications, notably skin defects and diminished muscle power [3,7,8].

**Figure 2:**
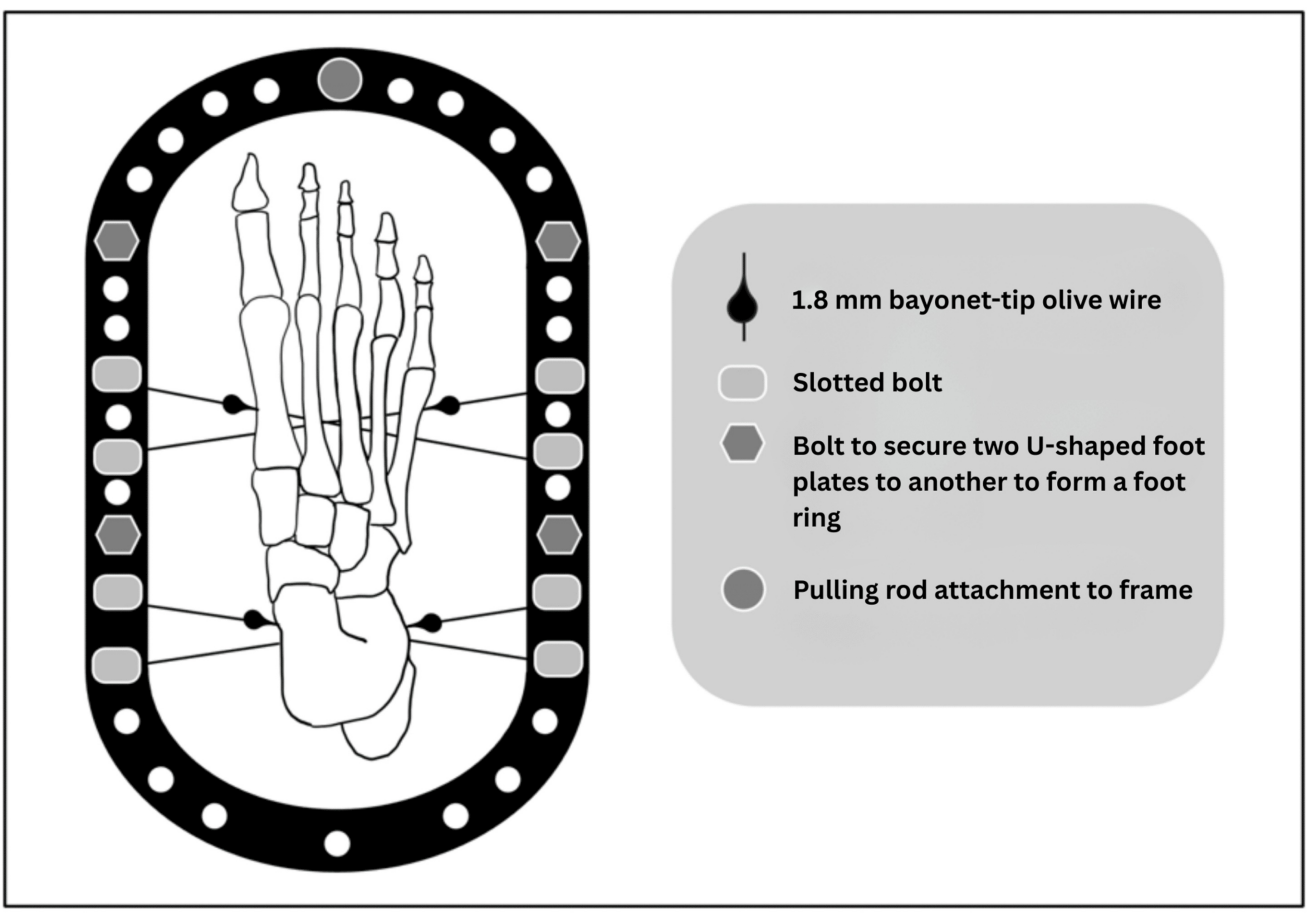
Schematic image showing the positions of the 1.8 mm bayonet-tip olive wires through the metatarsals and the calcaneus, and the position of the olives with the two connected U-shaped foot plates. Note that wires through the forefoot may not cross all five metatarsals but should cross at least three to ensure sufficient stability. Image Credit: Authors

The surgical procedure for the application of a foot plate on a pre-existing Taylor spatial frame is as follows: The patient with the existing equinus or equinovarus deformity is supine on the operating table. The entire leg, including the frame, is prepared using a standard surgical solution. To minimise the risk of neurovascular injury, we adhere strictly to the established safe corridors [9]. The neutral position of the foot is established by employing a flat surface tray, followed by applying two 1.8 mm bayonet-tip olive wires to the calcaneum and two 1.8 mm bayonet-tip olive wires to the forefoot. The standard position of the calcaneal wires is approximately 0.5 cm proximal to the line separating normal skin from the skin of the sole. This landmark facilitates the wires' placement at the calcaneus's plantar aspect. The two forefoot wires are applied similarly. Ideally, a forefoot wire should cross at least three metatarsals. The objective is to insert wires through the first to fourth metatarsals and, if feasible, the fifth one; however, this may prove challenging due to the arc of the forefoot. Upon the application of the wires, the foot plate is affixed so that it remains parallel to the tray. This configuration allows the patient to place their foot on the floor in the plantigrade position without the plate making contact with the floor (Figure 2).

Through diligent practice, we have concluded that there is no necessity to create hinges between the existing fine wire frame and the footplate, as the ankle joint will serve adequately as a hinge to enhance equinus and equinovarus deformity. In instances of equinovarus deformity, the pulling rod shall be affixed to the lateral side of the footplate to facilitate pronation and dorsiflexion of the foot. The position of the pulling rod may be adjusted as the deformity diminishes, as we intend to address the varus deformity initially, followed by the equinus deformity. Conversely, if only an equinus deformity is present, the position of the pulling rod on the foot plate is approximately central.

**Figure 3:**
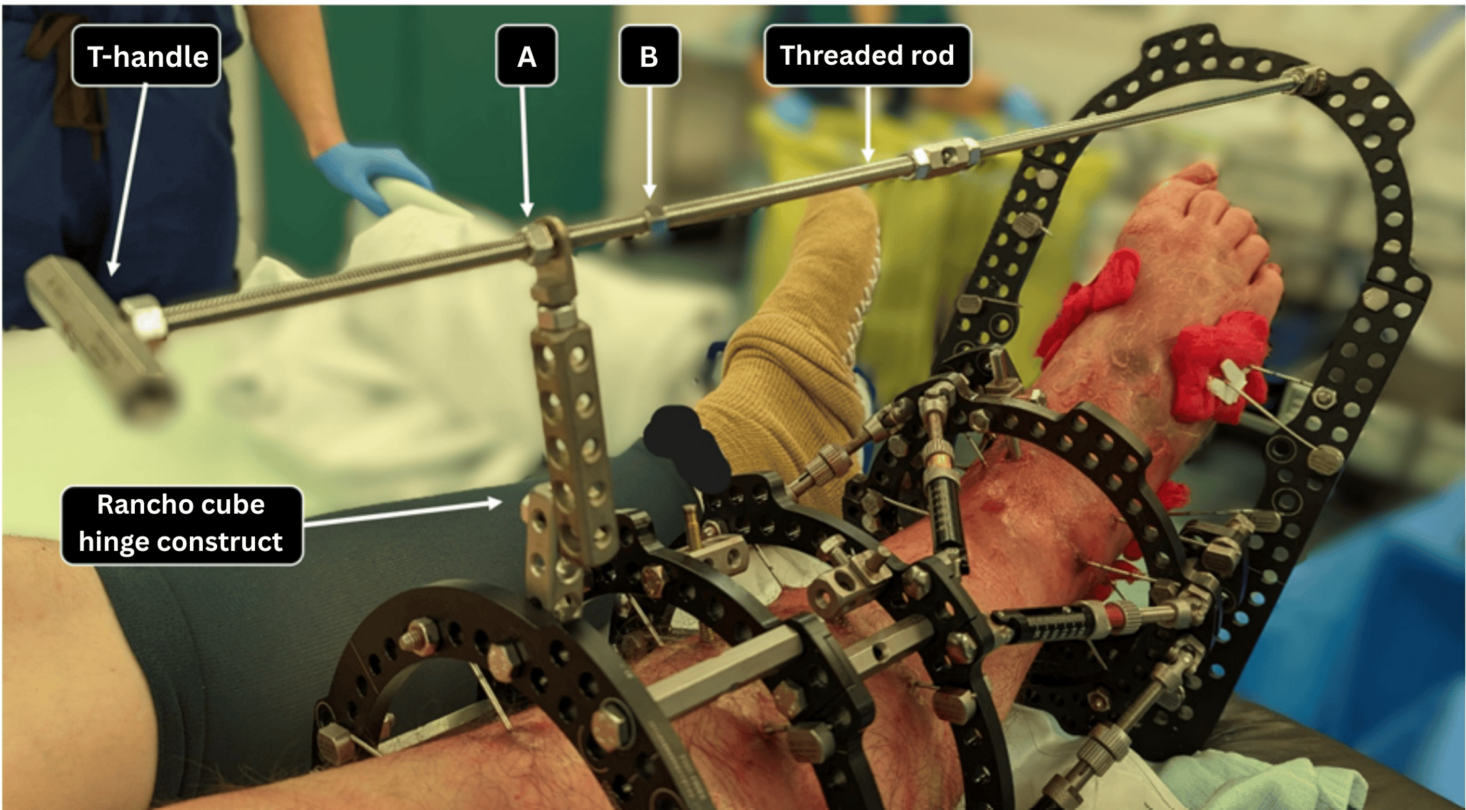
Photographic imaging in the operating theater after applying the foot plate on the right foot with the pulling rod connected just lateral to the foot's midline. Note the hinge mechanism utilizing rancho cubes at the two most proximal frame rings to facilitate movement of the pulling rod. The nuts “A” and “B” control the range of motion during the exercises and save the progress.

The pulling bar is affixed to the foot plate through a hinge supplied with the system and subsequently attached to the frame. Alternatively, the hinge may be constructed using various components of the system, typically the first or second ring from proximal to distal, with a rancho cube or another suitable connector. It is essential to ensure that the rod slides smoothly through the connectors, particularly when a substantial angle exists between the rod and the proximal rings. An additional T-handle may be positioned at the proximal end of the pulling rod, with nuts affixed to the threaded rod, to either monitor the improvement in the range of movement or to maintain a specific position of the foot. It is advisable to refrain from applying any forceful pull through the system during the application of the foot plate, as the action may induce significant pain, which could adversely affect progress (Figure 3).

**Figure 4:**
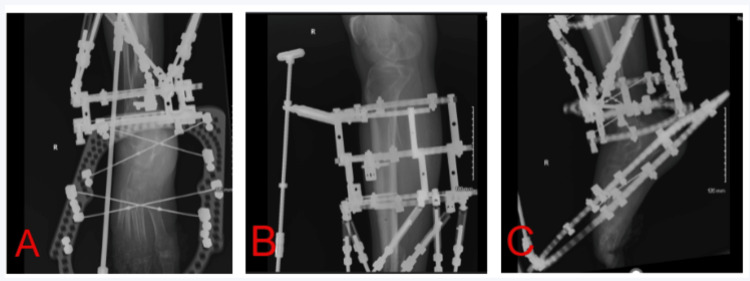
Direct postoperative X-ray imaging. A: Anteroposterior radiographic imaging of the distal right tibia and foot showing existing Taylor spatial frame, dual fine wire placements through the calcaneus and metatarsals, and a foot plate with a pulling rod. B: Lateral radiographic imaging of the right proximal tibia showing existing Taylor spatial frame with a rancho cube hinge construct connecting the frame to the pulling rod. C: Lateral radiographic imaging of the right distal tibia and foot following foot plate attachment to the existing Taylor spatial frame. Note the absence of any hardware connection between the foot plate and distal tibial Taylor spatial frame. The ankle is used as a natural hinge to facilitate dorsiflexion.

Postoperatively, patients are unable to bear weight on the construct, primarily due to the deformity of the foot and the footplate itself. Additionally, should patients attempt to walk on the foot plate, the forefoot wires are prone to rapid irritation, resulting in discomfort. Furthermore, the wires may also break and necessitate replacement (Figure 4).

**Figure 5:**
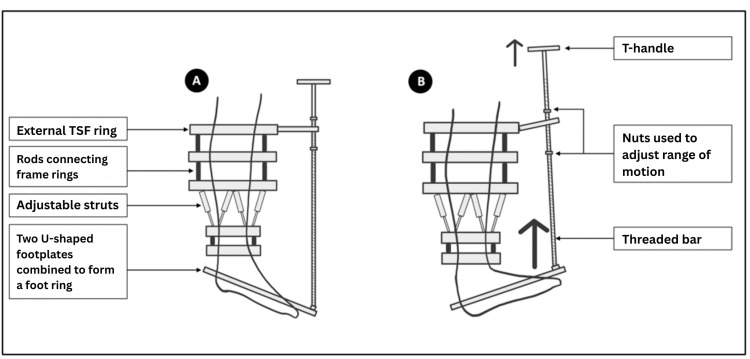
Schematic image showing the patient exercising by pulling up the T-handle to increase the foot dorsiflexion and the nuts' adjustments. A: Foot in plantarflexion. B: Patient pulling T-handle upward to bring foot into dorsiflexion. Note that the foot plate is not connected to the existing Taylor spatial frame, and the ankle joint is used as a natural hinge mechanism. TSF: Taylor spatial frame Image Credit: Authors

**Figure 6:**
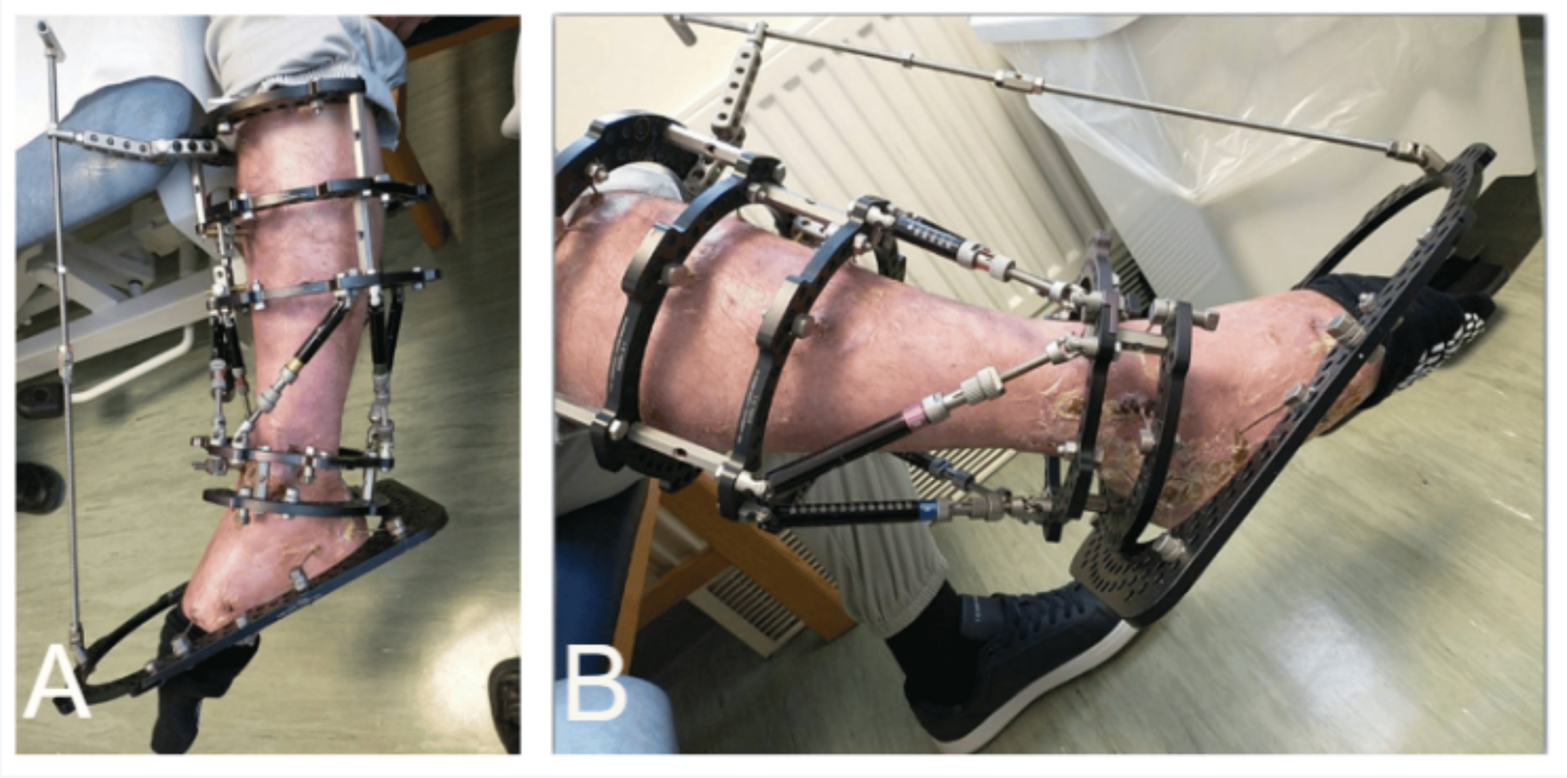
Photographic imaging of a fixed equinus deformity after the surgical application of the foot plate in a pre-existing Taylor spatial frame, showing the existing Taylor spatial frame and the foot plate with the T-handle extension on the right lower leg and foot.

The duration of treatment with a foot plate depends on the rate at which patients regain mobility. Patients are generally advised to exercise as frequently as possible, employing only gentle movements, and to cease any progression or pulling when pain is experienced (Figures 5-6).

On average, the treatment period lasts approximately three to four months. Upon achieving the neutral position of the foot, it is customary to hypercorrect it by a few degrees, as there is a tendency for recurrence of the contracture, particularly if the foot position is not protected following the removal of the foot plate [3,10]. The foot plate may be removed before the removal of the fine wire frame, or it may be removed simultaneously. Weight-bearing is reintroduced progressively following the removal of the foot plate, in conjunction with physiotherapy aimed at restoring and strengthening balanced muscle function of the lower leg, ankle, and foot.

## Discussion

In this technical report, we discuss a minimally invasive approach to enhancing equinus or equinovarus deformity of the foot following major injury or reconstruction of the lower leg. This technique represents a viable option for patients, particularly given that standard operating techniques, such as lengthening the tibialis posterior or Achilles tendon, cannot be performed due to spatial limitations when a fine wire frame is present or may result in a high complication rate in instances of compromised soft tissues in the leg [11,12]. Conversely, the technique described herein facilitates gradual improvement of the ankle joint and midfoot movement without significantly damaging these joints. Damage to the joints, specifically, refers to the tearing or dissection of the articular capsules due to contracture release procedures. In our experience, the primary disadvantage of the procedure is a relatively high recurrence rate, particularly if the foot is left unprotected following the removal of the footplate. In our practice, this is often accompanied by pin site irritations or infections, primarily affecting the forefoot wires, as these pins endure a long lever arm loading through the footplate and the pull rod. Furthermore, they may break and require replacement; however, such occurrences are infrequent. Lastly, there is the risk of neurovascular compromise secondary to iatrogenic injury. However, this risk can be minimized with careful pre-op planning, appropriate intra-op radio imaging, and keeping to the established safe corridors [9].

Finally, patient adherence to therapeutic protocols may reduce the likelihood of developing an equinus deformity in the first place, though evidence is limited [13,14]. It remains advisable to provide comprehensive guidance to patients with lower limb fine wire frames on the importance of weight-bearing and active participation in rehabilitation to mitigate the risks associated with prolonged immobilization and contracture development.

## Conclusions

Applying a foot plate with a fine wire frame constitutes a reasonable solution for the acquired equinus or equinovarus foot deformity in open tibia fractures and related inactivity or reverse bone transport for proximal tibial fractures. Should this technique be employed correctly preoperatively and postoperatively, it will enable patients to achieve a plantigrade foot without requiring any open surgical intervention, such as tendon lengthening or joint capsule releases. Complications associated with our approach are predominantly minor, such as pin site infections or contracture recurrence, and can be effectively managed using standard treatment protocols. A limitation of this report is that it is based on a limited number of cases, and clinical studies will be required to further validate the approach.

## References

[1] Widanage KN, De Silva MJ, Dulantha Lalitharatne T, Bull AM, Gopura RA (2023). Developments in circular external fixators: a review. Injury.

[2] Robinson JM, Mielke CH (2025 ). Ankle equinus. StatPearls [Internet].

[3] Dabash S, Potter E, Catlett G, McGarvey W (2020). Taylor spatial frame in treatment of equinus deformity. Strategies Trauma Limb Reconstr.

[4] Du H, He XT, Yin XH, Gu JM, Zhou YX, Yang J, Wu Y (2023). The gradual correction of adult severe rigid equinus deformity using minimal invasive U-osteotomy with Taylor spatial frame. Foot Ankle Int.

[5] DeHeer PA (2017). Equinus and lengthening techniques. Clin Podiatr Med Surg.

[6] Hsu RY, VanValkenburg S, Tanriover A, DiGiovanni CW (2014). Surgical techniques of gastrocnemius lengthening. Foot Ankle Clin.

[7] Wang C, Parekh SG, Mithani SK (2018). Management of Achilles tendon rupture complications. Instr Course Lect.

[8] Horsch A, Petzinger L, Deisenhofer J, Ghandour M, Klotz M, Renkawitz T, Putz C (2024). The impact of operative correction of equinus in cerebral palsy on gait patterns. Foot Ankle Int.

[9] Page RS (2001). Atlas for the Insertion of Trans-osseous Wires and Half-Pins. https://www.researchgate.net/publication/200162061_Atlas_For_The_Insertion_Of_Trans-osseous_Wires_And_Half-Pins.

[10] Emara KM, Allam MF, Elsayed MN, Ghafar KA (2008). Recurrence after correction of acquired ankle equinus deformity in children using Ilizarov technique. Strategies Trauma Limb Reconstr.

[11] Tabaie SA, Videckis AJ (2021). Achilles lengthening. J Pediatr Orthop Soc N Am.

[12] Dalton GP, Wapner KL, Hecht PJ (2001). Complications of achilles and posterior tibial tendon surgeries. Clin Orthop Relat Res.

[13] Harvey LA, Katalinic OM, Herbert RD, Moseley AM, Lannin NA, Schurr K (2017). Stretch for the treatment and prevention of contracture: an abridged republication of a Cochrane systematic review. J Physiother.

[14] Pawson JR, Church D, Fletcher J (2024). Rehabilitation techniques for adults undergoing external fixation treatment for lower limb reconstruction: a systematic review. Strategies Trauma Limb Reconstr.

